# Spatial Distribution of Genetic, Ploidy, and Morphological Variation of the Edaphic Steno-Endemic *Alyssum moellendorfianum* (Brassicaceae) from the Western Balkans

**DOI:** 10.3390/plants14020146

**Published:** 2025-01-07

**Authors:** Jasna Hanjalić Kurtović, Belma Kalamujić Stroil, Sonja Siljak-Yakovlev, Naris Pojskić, Adaleta Durmić-Pašić, Alma Hajrudinović-Bogunić, Lejla Lasić, Lejla Ušanović, Faruk Bogunić

**Affiliations:** 1 Institute for Genetic Engineering and Biotechnology, University of Sarajevo, Zmaja od Bosne 8, 71 000 Sarajevo, Bosnia and Herzegovina; jasna.hanjalic@ingeb.unsa.ba (J.H.K.); naris.pojskic@ingeb.unsa.ba (N.P.); adaleta.durmic@ingeb.unsa.ba (A.D.-P.); lejla.lasic@ingeb.unsa.ba (L.L.); lejla.usanovic@ingeb.unsa.ba (L.U.); 2 Ecologie Systématique Evolution, AgroParisTech, Centre National de la Recherche Scientifique (CNRS), Université Paris-Saclay, 91190 Gif-sur-Yvette, France; 3 Faculty of Forestry, University of Sarajevo, Zmaja od Bosne 8, 71 000 Sarajevo, Bosnia and Herzegovina; a.hajrudinovic@sfsa.unsa.ba (A.H.-B.); f.bogunic@sfsa.unsa.ba (F.B.)

**Keywords:** *Alyssum*, Balkans, diploids, endemic, diversity, structure, tetraploids

## Abstract

Polyploidy is a powerful mechanism driving genetic, physiological, and phenotypic changes among cytotypes of the same species across both large and small geographic scales. These changes can significantly shape population structure and increase the evolutionary and adaptation potential of cytotypes. *Alyssum moellendorfianum*, an edaphic steno-endemic species with a narrow distribution in the Balkan Peninsula, serves as an intriguing case study. We conducted a comprehensive analysis of genetic diversity and population structure across the species’ range, employing an array of genetic techniques (nuclear microsatellites, amplified fragment length polymorphisms, and plastid DNA sequences), flow cytometry (FCM), morphometry, and pollen analysis. The study reveals two genetic lineages: spatially distributed diploid and tetraploid cytotypes. Clear divergence between diploids and tetraploids was shown by AFLP, while plastid DNA sequences confirmed private haplotypes in each of the studied populations. Higher genetic diversity and allelic richness following the north-south pattern were documented in tetraploids compared to diploids, as indicated by nuclear microsatellites. Morphometric analysis via principal component analysis (PCA) and canonical discriminant analysis (CDA) did not reveal any divergence between diploid and tetraploid cytotypes. Nonetheless, a distinction in pollen size was clearly observed. The results suggest an autopolyploid origin of tetraploids from diploid ancestors. Despite the population fragmentation in a very small geographic range, these populations harbour high genetic diversity, which would allow them to remain stable if natural processes remain undisturbed.

## 1. Introduction

The plant diversity of the Balkan Peninsula presents significant challenges for botanists, particularly regarding endemic species, their origin, persistence and migrations during the interglacial periods, the identification of refugial areas, and their evolutionary potential in response to ongoing climate changes. The Balkan Peninsula is recognised as one of the important biodiversity hotspots and refugial areas in Europe, owing to its complex geological history, climatic fluctuations, heterogeneous topography, proximity of mountain ranges to the seas, and presence of various floristic elements [1,2,3,4]. These factors have shaped the spatial and genetic diversity of the Balkan flora [5,6,7], with over one-third being considered endemic [1,2]. The mild influence of Pleistocene climate oscillations localised glaciation to mountain peaks [8,9], facilitating inter- and intraspecific diversification in deglaciated areas in the Balkans [10,11]. Molecular studies published in the last two decades have revealed diverse evolutionary and phylogeographic patterns within different plant groups in the Balkans [12]. These patterns are linked to climate changes [13,14], geological history [15,16], ecological adaptations [17,18,19], long-term isolation in different refugia [20,21], and hybridisation [22,23].

In many of these plant groups, among others, polyploidisation is recognised as a key driver of diversification [22,23,24,25,26,27,28]. A classic example is the genus *Alyssum*, comprising 52 European species, of which 30 inhabit the Balkan Peninsula, and 16 are endemic [29,30,31]. The Balkans is considered the main centre of species diversity and endemism for the genus [30,31]. The diversity within *Alyssum* is generated by recurrent polyploidisation and hybridisation, followed by ecological specialisation and spatial isolation, suggesting a complex evolutionary history [20,32,33,34,35,36,37].

*Alyssum moellendorfianum* Aschers et Beck is a perennial steno-endemic species, edaphically specialised for dolomite substrate [38]. The species’ distribution range is extremely limited, covering less than 250 km^2^, with fragmented populations thriving on slopes of Mts. Prenj and Visočica along the Neretva River, the surroundings of Konjic and Glavatičevo in Bosnia and Herzegovina. It is a typical chasmophytic species, confined exclusively to dolomite rocky grounds and crevices [38]. The species is listed as endangered on the red list of vascular plants in the Federation of Bosnia and Herzegovina [39]. The main threats to *A. moellendorfianum* populations stem from the exploitation of dolomite sand and gravel, as well as fires and natural succession of vegetation. Knowledge of this steno-endemic plant remains incomplete, except for horological and vegetation data [38]. Recent phylogeographic and taxonomic studies of the genus in the Balkan Peninsula included two diploid populations of *A. moellendorfianum* and identified it as one of the five most morphologically and genetically distinctive species in the Balkans [30,31]. Siljak-Yakovlev et al. [40] revealed tetraploid *A. moellendorfianum* plants from another locality, indicating heteroploid structure of populations across the species’ range and a more complex population structure than previously recognised.

Given the national conservation concern for *A. moellendorfianum*, with identified threats to its populations in a very restricted distribution area, indications of different cytotypes, and a general lack of knowledge, a detailed understanding of various aspects of the species’ biology is necessary for developing an effective conservation strategy. In this study, we integrated different data types to investigate genetic diversity and differentiation and to analyse patterns of phenotypic variation across the species distribution range. These data should elucidate the patterns and processes governing the diversity of this steno-endemic plant. To achieve this, we employed a range of molecular techniques, including nuclear microsatellites, amplified fragment length polymorphisms (AFLP), and plastid DNA sequences, as well as flow cytometry (FCM), morphometry, and pollen analysis.

## 2. Results

### 2.1. Flow Cytometry Analysis

**Figure 1:**
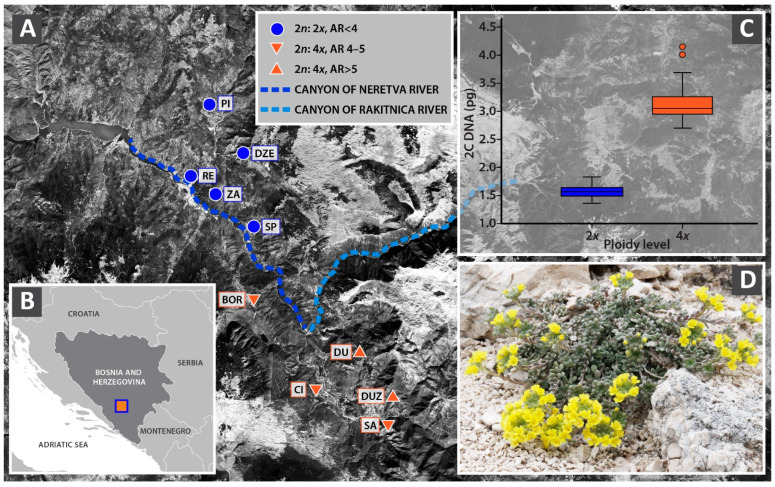
Sampling sites of *Alyssum moellendorfianum* populations analysed in the present study. (**A**) Cytotype and allelic richness (AR) distribution. Population codes correspond to those given in Table 1 (**B**) Species distribution range in Bosnia and Herzegovina (Western Balkans). (**C**) Box plots of absolute genome size for diploid and tetraploid cytotypes. (**D**) Habitus of *A. moellendorfianum* at DUZ locality; photo: F. Bogunić.

FCM analysis resulted in two distinct groups, with absolute genome size values corresponding to two ploidy levels: diploids and tetraploids (Table 1). The mean absolute (2C) genome size values for diploid and tetraploid cytotypes ranged from 1.472 to 1.644 pg and 2.922 to 3.233 pg, respectively. The mean monoploid (1C*x*) genome size values ranged from 0.731 to 0.822 pg. No significant differences were found in the mean 1C*x* values between the diploid and tetraploid cytotypes (*t* = 1.453, *p* ≤ 0.05). Geographically, five diploid populations had a northwestern distribution, whereas five tetraploid populations exhibited a southeastern distribution (Figure 1A). Each population was homoploid, irrespective of ploidy level.

### 2.2. Microsatellite Analyses

The average values of genetic diversity measures for five loci examined per population are presented in Table 2, with individual loci values per population available in Supplementary Table S1. In tetraploid populations, the average values of the number of detected alleles (A_N_), number of effective alleles (A_E_), allelic richness (AR), and observed heterozygosity (H_O_) were higher compared to diploid populations. The AR parameter followed a geographical cytotype distribution pattern, with values increasing from the northwest to the southeast (Figure 1A). The ratio of effective to detected number of alleles (R) was statistically significant (*p* < 0.05) for particular loci within tetraploid populations, as shown in Table S1, except for the BOR population, where no statistical significance was observed. In contrast, the effective and detected number of alleles ratio (R) was generally not significant among diploid populations, except for the PI population at the AP34461 locus (0.040; Table 2).

Analysis of genetic diversity in the total studied *A. moellendorfianum* sample revealed high frequencies of individual allelic variants at specific loci. The major allele frequency index (iMAF) showed statistical significance at three loci (Table 3).

The average values of the fixation index (F_ST_) suggested that 10.55% of the total genetic variation corresponded to differences among the 10 investigated populations. When comparing cytotypes, the average fixation index values of F_IT_, F_IS_, and F_ST_ were 0.3754, 0.3453, and 0.0461, respectively, implying that 4.61% of the genetic variation refers to differences between cytotypes. Furthermore, differences in genetic diversity parameters at the cytotype level were not statistically significant. These results were concordant with the analysis of molecular variance (AMOVA); the total variation was due to differences within populations (97.5%).

**Figure 2:**
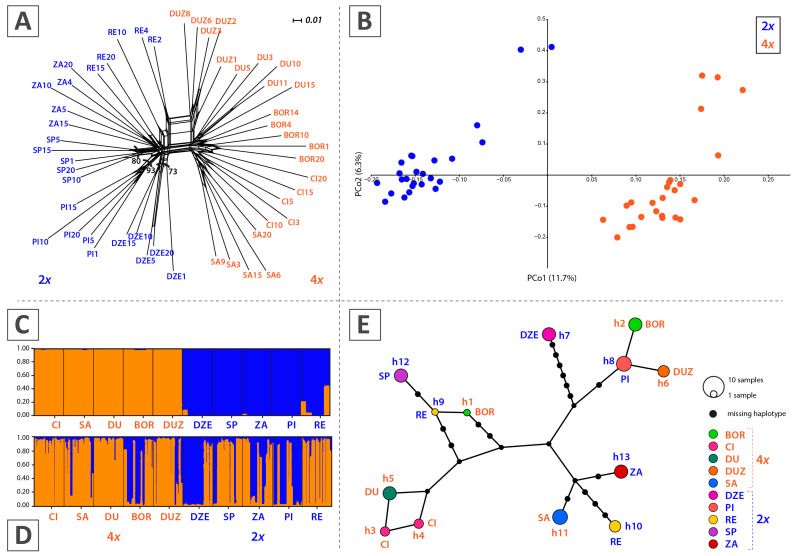
Genetic differentiation of the investigated populations of *Alyssum moellendorfianum*. (**A**) Neighbour-net diagram based on uncorrected P distances obtained from AFLP data. Numbers inside branches represent bootstrap values above 50% obtained by neighbour-joining analysis. (**B**) PCoA diagram based on Jaccard distances of AFLP data. (**C**) STRUCTURE analysis according to the AFLP matrix. (**D**) Proportion (%) to genetic clusters according to STRUCTURE analysis of microsatellite loci. (**E**) Statistical parsimony network (TCS network) based on identified haplotypes. Diploids are marked in blue and tetraploids in orange. Population codes correspond to those given in Table 1.

Pairwise F_ST_ indicated low rather than moderate differentiation (Supplementary Table S2), with the most significant differentiation observed between the diploid populations DZE and SP (pF_ST_ = 0.4289). The Bayesian clustering model and STRUCTURE analysis of microsatellite loci revealed two clusters (K = 2), with four tetraploid populations included in the second cluster based on the qI probabilities (probability of membership) higher than 74.9% (Figure 2D).

### 2.3. AFLP Fingerprinting

A total of 498 polymorphic fragments were scored for 50 individuals, with an error rate of 3.8%. The neighbour-net diagram revealed two clusters, separated by a short split, corresponding to diploid and tetraploid populations (Figure 2A). All individuals were grouped within their respective populations. Some higher bootstrap values were observed in three diploid populations: DZE (BS = 73%), SP (BS = 80%), and PI (BS = 93%) (Supplementary Figure S1). Tetraploid populations did not show a clear differentiation, except for the DUZ population, which was also not supported by significant bootstrap values (BS < 50%).

The principal coordinates analysis (PCoA) diagram displayed a similar pattern to the neighbor-net (Figure 2B), with the two groups clearly divergent along the first axis (PC1). The divergence of the DUZ population within the tetraploid cytotype was also evident (Figure 2A). STRUCTURE analysis indicated that the most likely number of clusters is K = 2 (Figure 2C). Both clusters were coherent and genetically homogeneous, consisting of diploids and tetraploids, respectively.

### 2.4. Chloroplast DNA Analysis

Amplification and sequencing of *rpoB-trnC* and *rpl32-trnL^UAG^* chloroplast intergenic spacers were performed for a total of 42 individuals, i.e., 84 sequences were used in further analysis (GenBank accession numbers PQ583517–PQ583600). The sequences of the analysed chloroplast intergenic spacers were concatenated for improvement signal over noise ratio. In the assessment of genetic diversity, only substitution sites were analysed. To avoid artificial increase in the diversity values, gaps were excluded. The concatenated matrix alignment consisted of 1666 bp positions. A total of 13 population-specific haplotypes, with 28 polymorphic sites, were found (Supplementary Table S4). No populations sharing a common haplotype were observed. Seven populations had only one haplotype; three populations had two different haplotypes detected (Figure 2E). Considering ploidy, six haplotypes were detected in diploid and seven in tetraploid populations. Differentiation of chloroplast haplotypes according to ploidy was not observed either through a statistical parsimony network (Figure 2E) or by neighbor-joining analysis based on pairwise distances across 10 populations (Supplementary Figure S2). Parsimony network revealed numerous unsampled/extinct haplotypes in the interior positions (Figure 2E).

### 2.5. Morphological Variation of Cytotypes

**Figure 3:**
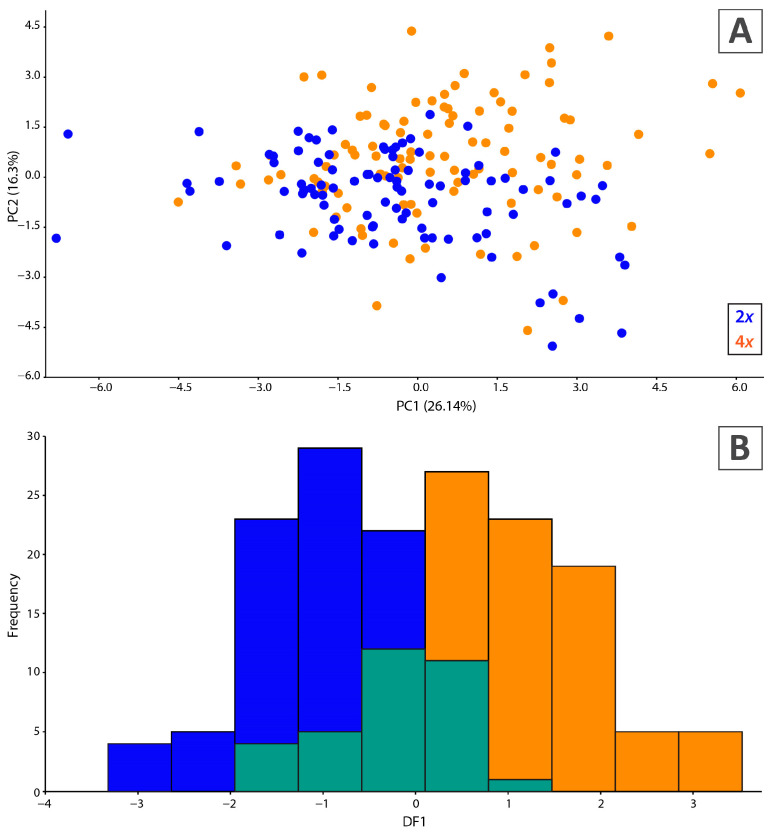
Morphological variation of cytotypes. (**A**) PCA ordination graph of studied individuals. (**B**) Distribution graph of individual canonical scores. Blue colour are diploids, orange are tetraplodis, and green are incorrectly classified individuals (diploids identified as tetraploids and vice versa).

PCA based on the individual dataset revealed an absence of morphological divergence between diploids and tetraploids. Most individuals intermixed regardless of ploidy level in the PCA scatterplot (Figure 3A). PCA generated five significant axes (eigenvalues > 1; two components are displayed) accounting for 66.1% of the total variance (PC1 = 26.1% and PC2 = 16.3%), with low to moderate correlations with the first and second axes (Figure 3A, Table 4). Similarly, PCA of population mean values (results not displayed) showed an overlap between diploid and tetraploid populations. Most morphological traits equally contributed both to PC1 and PC2 (Table 4).

The correlation values between all paired characters did not exceed r ≥ 0.85. The canonical discriminant analysis (CDA) model was significant (Wilks’ λ = 0.564, F_11,183_ = 12.81, *p* < 0.0001) and included 11 morphological characters. A canonical axis with a low eigenvalue (0.77) was extracted, explaining a total variance among the diploid and tetraploid cytotypes. The distribution of individuals along the first canonical axis displayed no gap on the distribution graph (Figure 3B). The following traits mainly revealed discrimination between the two groups (petal sinus and sepal length, partial Wilks’ λ values: 0.89 and 0.90, *p* < 0.0000) followed by trichome coverage on lower surface (partial Wilks’ λ = 0.94, *p* < 0.0008). The correlation coefficients of the traits related to the first discrimination function are displayed in Table 4. The results of classificatory discriminant analysis showed weak discrimination between the two groups, with 81.02% correct classification of all cases (*N* = 195), while 83% (*N* = 79) of the diploids were correctly determined vs. 79% (*N* = 79) of tetraploids.

### 2.6. Size and Viability of Pollen Grains

**Figure 4:**
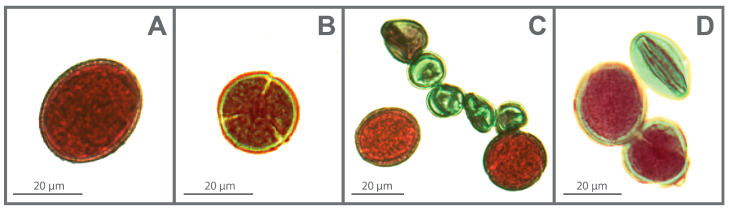
Meridian optical view (**A**) and polar view (**B**) of *Alyssum moellendorfianum* pollen grain; numerous micropollens from diploid Pirici population. (**C**) Sterile pollen grain (in green) from tetraploid Dudle population. (**D**) Bar = 20 µm.

The pollen grains of *A. moellendorfianum* are tricolpate and isopolar, with radial symmetry and reticulate exine ornamentation (Figure 4A,B). Both ploidy levels exhibited a subprolate shape of pollen (P/E = 1.14 for diploids and 1.20 for tetraploids).

Morphometric parameters for both the polar axis (P) and equatorial diameter (E) are detailed in Table 5. Pollen size increased significantly with ploidy level. In diploid populations, the mean pollen size was 29.16 µm for the polar axis and 25.55 µm for the equatorial diameter. In contrast, in tetraploid populations, the mean values were 35.1 µm for P and 29.29 µm for E. One-way ANOVA indicated significant differences between mean values of P and E traits for diploid and tetraploid populations (F_5,113_ = 94.1, *p* < 0.000, F_5,113_ = 85.3, *p* < 0.000). Tukey’s HSD tests identified differences between the diploid and tetraploid populations for both P and E traits, with significant differences also observed between the tetraploid population BO vs. SA, DU for the P trait, and between the tetraploid population BO vs. DU for the E trait.

Pollen viability varied insignificantly among the populations (F_9,49_= 1.97, *p* ≤ 0.067). The percentage of viable pollen grains ranged from 86.8 to 99% in diploid and from 91 to 99% in tetraploid populations (Supplementary Table S6). In diploid populations, aborted pollen grains primarily appeared as micropollen (3–13%, Figure 4C). This percentage was even higher in some individuals from the Pirici (40%) and Repovice (30%) populations, with an additional 7 and 10 large, well-constituted but sterile pollen grains observed (Figure 4D), respectively.

## 3. Discussion

### 3.1. The Spatial Distribution of Cytotypes

Our cytometric results accurately discriminated cytotypes whose ploidy levels were unambiguously inferred following 1C*x* values. The monoploid values were similar between diploids and tetraploids (Table 1), aligning with previous estimates [30,40]. Slight differences are frequently observed among different studies, potentially attributable to the use of different internal standards, fluorochromes, and either fresh or silica gel-dried plant tissues [41]. However, stable monoploid genome size values confirmed the reliability of our cytometric results.

Detailed screening of ploidy levels across a species range is of great importance due to the evolutionary impacts of polyploidy on life history traits of species [42,43,44,45,46]. The discovery of tetraploid populations in this study underscores the significance of conducting intraspecific ploidy screening, even in small geographic ranges. Most species within the *Alyssum montanum* L.—*A. repens* Baumg. complex in the Balkan Peninsula consist of di- and tetraploid cytotypes, with only a few exhibiting monoploidy [30,31]. Among the 112 Balkan populations investigated, only two were found to be heteroploid, namely in *A. austrodalmaticum* Trinajstić and *A. spruneri ‘graecum*’ [30]. The results indicate extremely limited or nonexistent gene flow, even in the aforementioned heteroploid populations where individuals of different cytotypes grow nearby [20,27]. However, neither heteroploid populations nor individuals with intermediate ploidy levels were detected in *A. moellendorfianum* within this study, despite the proximity of certain diploid and tetraploid populations (Figure 1A). Such a pattern suggests the absence of crosses between diploids and tetraploids of *A. moellendorfianum*, or at least none were detected. Mechanisms that strongly limit between cytotype hybridisation and maintain homoploid populations are indicated by the apparent prevalence of natural homoploid populations [47,48]. Additionally, a postzygotic reproductive barrier is most pronounced between diploid and tetraploid cytotypes [49]. The triploid block, caused by endosperm failure, is the most common reproductive barrier, hampering the formation of tetraploids from diploids during polyploidisation [50]. Although mechanisms to bypass the triploid block have been reported in various plant groups, this is clearly not the case in *Alyssum*.

The geographic distribution and clear separation of *A. moellendorfianum* cytotypes, as evidenced by AFLP, are intriguing. The distribution of cytotypes aligns with the position of Neretva and Rakitnica River canyons, where steep and narrow gorges form a natural geographical barrier between diploids in the northern part and tetraploids in the southern part of the distribution area (Figure 1A). The Neretva River valley is well known as a region with a phylogeographic split that inhibits gene flow and fosters intraspecific diversification in the Central Balkans [12]. Divergent environmental conditions, coinciding with the river’s position, occurred approximately 18,000 years ago during the Last Glacial Maximum (LGM) [51] due to shifting Adriatic Sea levels [13,52]. These geo-climatic changes affected the genetic diversity and geographic distribution of many Balkan plant species, including *Campanula austrodalmatica* D. Lakušić & Kovačić [51], *Edraianthus tenuifolius* A. DC. [52], *Salvia officinalis* L. [53], and *Cerastium decalvans* Schl. et Vuk [14]. All studied species have a much larger distribution area than *A. moellendorfianum*, but a similar scenario could be depicted.

The entire distribution of *A. moellendorfianum* populations is situated on the slopes of the high mountains Prenj and Visočica along the Neretva River, further surrounded by the mountains Crvanj, Bjelašnica, and Velež. While the Dinaric Mountains were less impacted by the Pleistocene glaciations [54], certain areas experienced significant glaciation during the LGM [55,56,57,58,59]. During the LGM, glaciers descended up to 900 m above sea level in these valleys, and ice fields on plateaus reached a thickness of 200 m [60]. Such conditions restricted altitudinal migrations of *A. moellendorfianum* in refugial areas during the LGM.

Additionally, the species’ edaphic specialisation on dolomite substrate restricted horizontal migrations due to fragmented habitats, such as dolomite rocky grounds and crevices [61]. Consequently, the range expansions of *A. moellendorfianum* cytotypes were likely hindered to remote areas more by dolomite substrate specialisation than by climatic oscillations during the Pleistocene. Thus, the species’ distribution area primarily depends on the availability of appropriate habitats [61], which are further influenced by climate factors, fires, and human impact.

Moreover, the low dispersal capacity, restricted pollen, and seed dissemination of many *Alyssum* species influence the prevalence of endemism and spatial structuring of genetic variation [62]. Gravity and epizoochory, indirectly facilitated by human activities, are the primary factors in seed dispersal within the genus [36]. In the absence of specific seed adaptations favouring greater dispersal capacity, geographic barriers can significantly reduce gene flow [36,62]. Consequently, gene flow between *A. moellendorfianum* populations or cytotypes is further impeded due to the isolated habitats. The presence of private haplotypes within each population and the absence of heteroploid populations suggest barriers to seed migration, indirectly limiting gene flow between diploid and tetraploid cytotypes.

### 3.2. Genetic Diversity and Structure of Alyssum moellendorfianum

In *A. moellendorfianum*, all examined diversity parameters were higher in tetraploid populations based on microsatellite markers. The A_E_ represents the allele’s contribution to the overall heterozygosity. The proportion of alleles that do not contribute to the overall genetic diversity of populations is indicated by a statistically significant difference in the ratio of A_E_ to A_N_ (R) [63]. A smaller percentage of alleles contribute to the overall genetic diversity in tetraploids, according to the R-ratio results considering the overall sample (Supplementary Table S1). This could be due to selection pressure or genetic drift within small, closed populations. Populations exhibiting a diploid cytotype have a higher proportion of alleles that contribute to genetic diversity. The MAF index results (Table 3) also indicate the presence of selective pressure on the analysed loci, as some alleles tend toward fixation, leading to the loss of less frequent alleles [64].

The heterozygosity results showed that these isolated populations within a small distribution area still exhibit high diversity. Allelic richness and heterozygosity are direct indicators of average individual fitness, though their significance varies from ecological and evolutionary perspectives [65,66]. Short-term responses to selection and adaptation are directly influenced by heterozygosity measures, while long-term responses favour populations with higher allelic diversity due to their greater adaptability and evolutionary potential [67]. Allelic richness represents the evolutionary strength of a population in adapting to potential environmental changes, as this parameter includes the share of unrelated adaptive potential through neutral alleles [68]. The diploid population DZE exhibited the lowest observed and expected heterozygosity and low allelic richness, indicating a narrow range of adaptation to environmental stressors. In fragmented habitats, allelic richness and heterozygosity are often reduced; however, these reductions are strongly correlated with the timing of changes, the reproductive system, or the unique response of each plant species [69]. According to the most recent population census from 2013, roads leading to populated areas in Konjic municipality pass through habitats of *A. moellendorfianum* populations, suggesting that anthropogenic factors, in addition to fragmentation, may affect the genetic diversity of this species. 

Analysis of the genetic divergence and structure of *A. moellendorfianum* revealed a notable pattern. The studied populations were grouped into two cytotype-distinctive clusters based on AFLP markers. Compared with tetraploids, which exhibited low divergence, three diploid populations showed more significant divergence, as supported by bootstrap values (BS > 50%). The observed pattern likely results from the longer mutual isolation of diploid populations. Tetraploid populations may have a more recent origin, with insufficient time for divergence processes to deeply impact their structure, similar to *A. repens* populations [35]. Additionally, *Alyssum* species studied in the Balkan Peninsula were grouped into multiple clusters, with only a few showing higher bootstrap values, indicating distinct genetic groups [30]. The highest divergence among Balkan species was recorded in di- and tetraploid populations of *A. austrodalmaticum* (BS = 80%) and diploid populations of *A. vernale* Kit. ex Hornem. (BS = 88%), while eight species, including populations of *A. moellendorfianum*, were supported with BS values < 50%. These populations were grouped with high mountain diploids and tetraploids of *A. bosniacum* Beck., supporting their designation as sister species [30]. A similar pattern of genetic diversity and population structure in relation to bioclimatic variables was observed for *Helichrysum italicum* (Roth) G. Don fil. [19], *Salvia officinalis* [53], and Cerrado plant species [70], indicating a recent development of local adaptation and divergence processes.

Variability in chloroplast intergenic spacers was demonstrated in research on the species complex (*A. montanum–A. repens*) of the Apennine Peninsula (48 haplotypes/16 populations) [71] and the Balkan region (117 haplotypes/112 populations) [30]. Similar variability was observed in the group *A. ovirense* A. Kern/*A. wulfenianum* Bernh. ex Willd. (10 haplotypes/10 populations) [32] and the diversification of *A. montanum* (87 haplotypes/113 populations) [33]. However, the interspecies variation from the study by Španiel et al. [30] was significantly greater, with *A. moellendorfianum* among five species recording highly divergent haplotypes. Overall, patterns of chloroplast DNA variation indicated numerous missing ancestral haplotypes, confirming the complex evolutionary history of this group in the Balkans. A general trend toward the appearance of low genetic structuring is evident due to recent rapid diversification followed by frequent reticulation and parallel polyploidisation. This has led to the formation of a complex of species with pronounced ancestral polymorphism and low genetic and morphological differentiation, resulting in incomplete lineage sorting [37].

### 3.3. From Diploids to Tetraploids: Challenges in Polyploid Origin Reconstruction

Reconstructing the polyploid origin is particularly challenging due to the processes of recomposition and recombination of the polyploid genome, extinction of parental lineages, multiple origins, loss or conversion of genes, and epigenetic mechanisms, particularly in cases of species complexes, despite advances in methodology [27,35,37]. Autopolyploidisation is likely the process forming *A. moellendorfianum* tetraploids. The stability of genome size within both cytotypes, spatial distribution, and differentiation of di- and tetraploid cytotypes according to AFLP and microsatellite analyses suggest autopolyploid origin. This study aligns with Soltis and Soltis [72] and Soltis et al. [42], who found that autopolyploids exhibit higher values of heterozygosity and allelic richness compared to their diploid ancestors. A recent study by Španiel et al. [37] employing high-throughput sequencing of the *Alyssum* species complex discovered low genetic divergence, hybridisation, and ILS as common occurrences within the genus. Phylogenetic analysis of diploid species’ nuclear genes revealed hybridisation, with *A. moellendorfianum*’s genome containing γ = 36% of *A. bosniacum*’s genetic material. However, nuclear phylogeny conclusions were not supported by phylogenetic analysis of the plastid genome, likely due to the random sorting of ancestral polymorphism within chloroplast genomes [37]. Indeed, genome introgression may have occurred as high mountain populations of *A. bosniacum* from Prenj and Visočica surround the range of *A. moellendorfianum*. These areas, with abundant traces of glacial processes, likely facilitated the altitudinal movements of *A. bosniacum* toward lower altitudes in response to climatic oscillations, allowing them to survive within refugia during the LGM. The complete reconstruction of the tetraploid *A. moellendorfianum*’s origin, along with evidence of genome introgression from *A. bosniacum*, is yet to be determined.

In contrast, allopolyploidisation is more prevalent in the *A. montanum–A. repens* complex in the Balkan Peninsula, according to Španiel et al. [30]. Autopolyploids have been identified in *A. fastigiatum* Heywood in Spain and *A. gmelinii* Jord. & Fourr. in Central Europe, based on genetic relatedness and lack of morphological differentiation between di- and tetraploids [33,71]. Multiple polyploidisation events during the formation of *A. repens* tetraploids could not distinguish whether they are of auto- or allopolyploid origin [35]. The origin of the *A. repens* allotetraploid group is associated with the parental genomes of the Balkan populations of *A. vernale*, *A. moellendorfianum*, and *A. bosniacum*, underscoring the extensive hybridisation among the genus’s representatives from the Balkans and the challenging reconstruction of polyploid origins due to hybridisation at both diploid and polyploid levels. The main factors influencing the formation of polyploids within the genus include the parallel formation of allopolyploids from common pairs of diploid species, as well as autopolyploid lineages and species from a common ancestor with subsequent range fragmentation and consequent genetic differentiation due to long-term isolation and adaptation within different glacial refugia [20,35,37]. It remains unclear how much of the recent cytotype divergence is due to polyploidisation compared to genetic divergence following the emergence of polyploidy [73].

Our PCA and CDA morphometrics-based approach did not confirm morphological divergence between the cytotypes. The absence of morphological differentiation between diploid and tetraploid *A. moellendorfianum* cytotypes contrasts with the clear separation observed in the AFLP data (Figure 2A). Polyploidisation is known to have a profound effect on biodiversity at different scales [74,75], inducing phenotypic and physiological changes [76,77], providing adaptive potential and enabling ecological divergence [78]. It can also modify reproductive modes [79]. Given the small distribution area and the ecologically similar habitats across the same elevations where both cytotypes thrive, significant phenotypic differentiation would be unexpected. This pattern is common in autopolyploids, where morphological differences are often absent or ambiguous, even among a series of polyploids [80,81,82,83,84]. Additionally, we did not observe any obvious ecological differentiation that could be linked to the morphological divergence of the cytotypes. Thus, we refrain from recognising the cytotypes as distinct taxonomic entities; additional studies, including a more detailed investigation of the relationship between ploidy distribution, morphological patterns, and microclimate within the area to determine the potential niche differentiation of cytotypes given their spatial separation, are needed.

Unlike morphology, polyploidy influenced pollen size by increasing the ploidy level in *A. moellendorfianum*. The relationship between polyploidy and increased pollen size has been demonstrated in various plant groups [85,86,87,88,89,90], including representatives of the Brassicaceae family [91]. However, the extent to which polyploidy impacts the adaptive and functional significance of increased pollen size in the tetraploid cytotype of *A. moellendorfianum* remains unclear. Baser et al. [92], Al-Blesh and Al-Joaany [93], and Erden and Menemen [94] studied the morphology and size of pollen grains in the genus *Alyssum*. The morphological type was consistently either prolate or subprolate, depending on the P/E ratio, although these studies lacked data on chromosome number or ploidy level. The pollen size of *A. simplex* Rudolphi (P = 35.97 µm, E = 26.16 µm), reported by Erden and Menemen [94] from Turkey, closely approximates that of *A. moellendorfianum*. These values likely correspond to the tetraploid cytotype of this species, similar to *A. moellendorfianum*. In fact, two ploidy levels were reported for *A. simplex* diploids from Spain [95] and tetraploids from Iran [96] and Morocco [97]. On the other hand, pollen viability in *A. moellendorfianum* was high, except in certain individuals belonging to the Borci, Dzepi, and Pirici populations, which produced numerous micropollen (Figure 4C) or sterile pollen (Figure 4D), indicative of meiotic irregularities. Nonetheless, population-level pollen viability results suggest regular microgametogenesis in both cytotypes. Further research should focus on meiotic processes and in vitro and in vivo pollen germination and experimental crosses between diploid and tetraploid cytotypes.

### 3.4. Implications for Conservation

Data on genetic diversity are crucial for developing an effective management and conservation strategy. Over 90% of human-caused extinctions affect species unique to a single country, underscoring the critical role national conservation strategies play in achieving global biodiversity goals [98]. Many endemic species are highly vulnerable, emphasising the need for targeted conservation efforts at both national and international levels [99,100,101,102].

Populations of endemic species, due to their limited distribution or isolation, are particularly susceptible to anthropogenic pressures or environmental changes [99,103]. The habitats of *A. moellendorfianum* are characterised by high slopes and loose dolomite substrate. One key measure to prevent soil erosion has been afforestation with black pine, a practice ongoing for over 100 years. However, this also facilitates forest succession, potentially displacing this species out of its habitats. Frequent, human-induced fires pose another risk, though their impact on population stability is still under research. Due to the shallow vegetation cover in these habitats, fires tend not to cause major damage. During sample collection for morphometric analyses in April 2023 at the Dužani locality, which had burned three years prior (Voloder F., pers. communication), the abundance of *A. moellendorfianum* individuals was observed to be similar to that in other unburned localities (Hanjalić J., Bogunić, F., field observation). Additionally, using dolomite substrate for separating gravel and sand as a construction material poses another risk to the species. Although faced with numerous pressures, the results of this study indicate a high level of allelic richness and heterozygosity, which enables the stability of populations in current conditions. Long-term monitoring and detailed analysis of local spatial structures are essential for maintaining genetic diversity [104]. Future monitoring of genetic diversity should include at least two populations of each cytotype.

Protecting habitats is crucial for conservation of *A. moellendorfianum*. The “Vrtaljica” protected area serves as a significant habitat for the diploid cytotype. The genetic diversity of diploid population DZE suggested limited adaptability to environmental stressors; thus, it needs to be included in further monitoring. Similar protection is necessary for other habitats, including those inhabited by the tetraploid cytotype. For *A. moellendorfianum*, balancing feasible conservation measures with the need for afforestation of erodible habitats is essential. Current efforts to establish an educational trail in Vrtaljica may enhance protection of *A. moellendorfianum* subpopulations by raising public awareness. Continuous education on the importance of endemic species and biodiversity could help reduce human impact on these populations. In situ conservation is recommended as the most effective approach, as it safeguards entire gene pools within natural habitats, strengthening population resilience through habitat protection [105]. When combined with ex situ strategies, such as seed banking and establishing collections in botanical gardens, these measures can maximise the preservation of *A. moellendorfianum*.

## 4. Materials and Methods

### 4.1. Plant Material

We sampled a total of 10 populations of *A. moellendorfianum* across its entire distribution range during 2017 and 2019/2020 (Figure 1A,B). Detailed information on geographic locations and the number of analysed individuals per population is provided in Table 6. Fresh leaves were collected, silica-dried, and used for flow cytometric measurements and DNA extraction. Flowering shoots were herbarised and used for morphometric analyses. Voucher specimens have been deposited in the herbarium of the National Museum of Bosnia and Herzegovina (SARA), Sarajevo, Bosnia and Herzegovina.

### 4.2. Genome Size and Ploidy Level Determination

Absolute genome size and ploidy level determination were performed using FCM, following the protocol described by Bourge et al. [41]. Leaves from the samples and internal standard (~30 mg, *Lycopersicon esculentum* Mill. cv ‘Roma’, 2C = 1.99 pg) [106] were simultaneously chopped in 1 mL of Gif nuclear isolation buffer (GNB: 45 mM MgCl_2_, 30 mM sodium citrate, 60 mM MOPS (pH = 7), 1% (*w*/*v*) PVP 10,000 (pH = 7.2), 0.1% (*w*/*v*) Triton X-100, 5 mM sodium metabisulphite [41]) in a plastic Petri dish. Suspension of nuclei was filtered through 50 μm pore size nylon mesh and saturated on ice for 5 min. Approximately 2000–5000 nuclei stained with propidium iodide (50 μg/mL final concentration, PI, Sigma Aldrich, St. Louis, MO, USA) were registered using the CytoFLEX S cytometer (Beckman Coulter-Life Science, Indianapolis, IN, USA, excitation 561 nm, 30 mW; emission through a 610/20 nm band-pass filter). The 2C DNA values were calculated based on a linear relationship between the fluorescence signals of the unknown samples and the known internal standard. The fluorescence histograms were analysed using Kaluza software ver. 2.1 (Beckman Coulter, Brea, CA, USA). Ploidy levels were determined by comparing the absolute genome size with the known chromosome count (DNA ploidy level) [29]. Standard statistical parameters including mean value, coefficient of variation, standard deviation, and minimum and maximum values, were calculated. The mean values of monoploid genome size between diploid and tetraploid cytotypes were compared using a *t*-test.

### 4.3. DNA Extraction and Molecular Analysis

Total genomic DNA was extracted from 20 mg of silica-gel dried leaves using the CTAB organic extraction protocol [106,107]. The samples were prepared using Retsch TissueLyser (Retsch GmbH, Haan, Germany). The quality of the extracted DNA was assessed through horizontal electrophoresis on a 1.5% agarose gel in 1 x SB buffer (pH 8) [108] after staining with Midori Green Advance (Nippon Genetics Europe GmbH, Düren, Germany). Gel images were captured using the Vilber Fusion documentation system (Vilber Lourmat, Eberhardzell, Germany). All PCR-based procedures were carried out using GeneAmp PCR System 9700 (Applied Biosystems, Foster City, CA, USA) and Alpha Thermal Cycler (PRCmax, Cambridge, UK) machines. Amplicons were detected with an ABI PRISM 3500 Genetic Analyser (Applied Biosystems). Capillary electrophoresis was performed for 40 min with the following conditions: 50 cm long capillary, POP-7^TM^ polymer, 15.0 kV, and 60 °C. Allele sizes were calculated using the internal size standard GeneScan 500 LIZ (Applied Biosystems). Electropherograms were analysed using GeneMapper v5 software (Applied Biosystems).

### 4.4. Microsatellite Genotyping

Eight nuclear microsatellite loci developed for *Odontarrhena serpyllifolia* (Desf.) Jord. & Fourr (syn. *Alyssum serpyllifolium* Desf) from Sobczyk et al. [109] were tested for cross-amplification (Supplementary Table S3). Five loci were steadily and consistently amplified and used in further analyses (AP31733, AP31679, AP31640, AP34461, and AP10368). We optimised two multilocus reactions and one PCR reaction.

The total volume of multilocus PCR reactions was 15 µL with 0.2 µM primers AP31733 and AP31679 (mix 1), 0.25 µM primer AP10368 and 0.15 µM primer AP31640 (mix 2), 0.2 mM dNTPs, TaqNovaHS buffer 1x (Blirt, Gdansk, Poland), 1.5 mM (mix 1) or 2.0 mM (mix 2) MgCl_2_, 1U/µL TaqNovaHS DNA polymerase (Blirt), and 1 µL gDNA template. The volume of the single PCR reaction was 10 µL with 0.2 µM primer AP34461, and the other chemical conditions corresponded to mix 2.

The PCR thermal cycling protocol included an initial denaturation step at 95 °C for 3 min followed by 30 cycles of denaturation at 95 °C for 30 s, annealing at 55 °C for 30 s, and elongation at 68 °C for 1 min. The final step was at 68 °C for 5 min. The products from the single PCR reaction were combined with those from mix 2 for fragment analysis.

Genotypes were analysed using SPAGeDi ver. 1.5d software [110]. Expected and observed heterozygosity was calculated according to Nei [111]. The ratio of effective and detected number of alleles (R) and the most common allele frequency index (iMAF) were analysed using the R scripts ALRATIO [63] and MAF [64]. Genetic variability distribution was estimated using AMOVA, and differentiation was calculated as described by Weir and Cockerham [112]. The Bayesian model in the STRUCTURE program was used for the population structure analysis [113]. The POLYSAT [114] within the R [115] was used to generate the input document for STRUCTURE. The Structure Harvester [116] was used to estimate the number of clusters (ΔK) [117]. The parameters were 100,000 burn-in generations, 1,000,000 MCMC replications, and 5 iterations for each K-defined number of clusters (K = 1–10).

### 4.5. AFLP Fingerprinting

AFLP fingerprinting was performed according to the methodology described by Vos et al. [118] with modifications that enabled fluorescent capillary electrophoresis. The digestion, adapter ligation, and pre-selective amplification were performed following the protocol described by Trybush et al. [119]. Selective amplification included primer pair combinations as follows: (1) FAM-EcoRI-A-GAG/MseI-CAC; (2) VIC-EcoRI-A-ACG/MseI-CAC; and (3) NED-EcoRI-A-TGC/MseI-CAC. PCR reactions in a total volume of 10 µL contained 0.7 µM fluorescently labelled EcoRI selective primer and unlabelled MseI primer, followed by 2.0 mM dNTPs, TaqNovaHS buffer 1x (Blirt), 1.5 mM MgCl_2_, 1.5 U/µL TaqNovaHS DNA polymerase (Blirt), and 1 µL of 10-fold diluted pre-selective PCR product as the template. The following thermal protocol was applied: initial denaturation at 94 °C for 3 min, 12 cycles of denaturation at 94 °C for 30 s, annealing at 65 °C for 30 s with a decrease of 0.7 °C/cycle, and elongation at 72 °C for 1 min, followed by 23 cycles of denaturation at 94 °C for 30 s, annealing at 56 °C for 30 s, elongation at 72 °C for 1 min, and final elongation at 60 °C for 30 min. In the following step, the samples were denatured for 5 min at 95 °C and immediately placed on ice.

Amplified fragments between 100 and 500 bp were transposed into a binary data matrix and scored as having present (1) or absent (0) peaks. The minimum relative intensity of the peak detection threshold was set to 100 rfu. The reproducibility of the AFLP profiles was tested using five replicated samples (10% of the final dataset). The error rate was calculated as the ratio of fragment differences to the total number of comparisons [120]. The Nei-Li genetic distance was used to assess the relationships between the examined populations [121]. Based on the matrix of uncorrected P distances from SplitsTree ver. 4.12 [122], a bootstrapped (2000 pseudoreplicates) neighbour-joining analysis was carried out using TREECON 1.3 b software [123] and visualised with a neighbour-net diagram [124]. PCoA based on Jaccard distance was conducted using the PAST ver. 3.14 software [125]. Population structure analysis based on AFLP data was performed using the STRUCTURE programme [113] with the same parameters as used for microsatellite loci.

### 4.6. Chloroplast DNA Analyses: rpoB-trnC and rpl32-trnL^UAG^ Intergenic Spacers

Two intergenic spacers of chloroplast DNA, rpoB-trnC [126] and rpl32-trnLUAG [127], were used for plastid haplotype detection. PCR reactions in a total volume of 35 µL contained 0.2 µM primers, 2.0 mM dNTPs, TaqNovaHS buffer 1x (Blirt), 1.5 mM MgCl_2_, 5 U/µL TaqNovaHS DNA polymerase (Blirt), and 1 µL gDNA template. The temperature regime of the PCR reactions included initial denaturation at 80 °C for 5 min, followed by 35 cycles of denaturation at 95 °C for 1 min, annealing at 50 °C for 1 min, elongation at 65 °C for 1 min, and one final cycle of elongation at 65 °C for 5 min. Purification and sequencing were performed by Eurofins Genomics (Ebersberg, Germany). Sequence identity was confirmed using the identity and similarity index within GenBank on the NCBI platform with the BLAST tool [128] for local alignments and sequence homology. Nucleotide sequences were optimised using BioEdit [129]. Multiple sequence alignment was performed using ClustalX 2.0 [130] under standard settings (Gap open/Gap extension penalty: 10.0/0.2. Weight Matrix: Gonnet). The dataset for further analyses was created based on PQ583555 for rpl32-trnLUAG and PQ583565 for rpoB-trnC sequences. The output was used to determine plastid haplotypes using MEGA6 software [131]. DNA polymorphism analysis of chloroplast sequences was conducted using the DNAsp ver. 6.12.03x64 [132]. PopART v1.7 [133] was used to infer a statistical parsimony network (TCS network) based on the detected haplotypes (concatenated sequences of rpl32-trnL and rpoB-trnC) and following Clement et al. [134].

### 4.7. Morphometric Analyses

Seventeen floral and vegetative morphological traits were analysed following Španiel et al. [31]. The examined characters were quantitative except for trichome coverage on the upper and lower surfaces of the stem leaves (semiquantitative, 0–3). Measurements were performed on herbarium specimens using SZ61 stereo microscope (Olympus, Tokyo, Japan) and CellSens (Ver.4.1) imaging software (Olympus). The floral and vegetative measurements (Supplementary Table S5) were averaged to construct two datasets: individual and population matrix data. The individual data matrix was based on means of individual plants, and the population matrix was based on all measurements at the population level (results are not displayed). We performed PCA to evaluate the morphological variation between diploids and tetraploids. Also, we performed CDA to determine the most distinguishing morphological traits and to classify individual plants into a priori specified groups (diploids vs. tetraploids). All data were standardised before analyses due to different scales of trait scoring (quantitative and semiquantitative scoring) [135]. PCA was based on the correlation matrix of the measured traits and the axes corresponding to principal components with eigenvalues > 1. CDA was computed based on the Mahalanobis distances of 11 variables included in the model (see Results). The histogram of frequency of individual discriminant function scores was used to display the distribution of analysed individuals following the group assignment (diploids and tetraploids). Next, CDA was used to determine the proportion of individual trees to be assigned to cytotype group by cross-validation procedure. Pearson correlation coefficients were calculated to determine highly correlated character pairs (r > 0.85) since these could distort the results of discriminant analysis [136]. All analyses were performed in PAST ver. 3.14 software [125] and Statistica ver. 8.

### 4.8. Pollen Grain Measurements and Viability Estimation

Pollen grains obtained from herbarium specimens were prepared according to Wodehouse’s method [137] and mounted in glycerin jelly, allowing them to fully hydrate for at least three weeks. The polar axis (P) and equatorial diameters (E) were measured in the meridian and equatorial views for 120 pollen grains (20 per population), i.e., 60 grains for each ploidy level. All measurements were performed on well-formed pollen grains in meridian optical view under 600× magnification (using a 40× objective lens and 15× ocular micrometre), using a M20 microscope (Wild, Heerbrugg, Switzerland). A one-way ANOVA was applied to analyse the mean P and E values between the cytotypes. Additionally, post-hoc Tukey HSD test was conducted to determine differences among the populations.

Alexander’s stain was employed to assess pollen viability [138] for each population, surveying 100 pollen grains per individual. Pollen grains were classified as viable (well-stained) or non-viable (shrunken and unstained, or aborted as micropollen). Pollen quality was expressed as the percentage of viable grains. ANOVA was conducted on Z-transform percentage data.

## 5. Conclusions

Our molecular data revealed complex patterns of diversity and structure in *A. moellendorfianum*, characterised by two cytotype clusters, considerable variation in chloroplast DNA, and evidence of incomplete lineage sorting. These findings illustrate an intricate evolutionary history driven by polyploidisation, limited gene flow, and recent diversification within a small distribution range. While genetic evidence, such as AFLP and microsatellite analyses along with pollen analysis, confirms differentiation between di- and tetraploids, the absence of clear morphological divergence aligns with the common pattern seen in autopolyploids. This study underscores the challenges of reconstructing polyploid origins, suggesting that further research into microclimatic factors and spatial distribution patterns may provide additional insights into the ecological dynamics and potential niche differentiation of *A. moellendorfianum* cytotypes.

This study is valuable from a conservation perspective for developing informed conservation strategies for this endangered, steno-endemic species. To effectively conserve *Alyssum moellendorfianum*, a multifaceted approach is required, prioritising habitat preservation and legal protection. Addressing anthropogenic pressures, such as afforestation and fire management, alongside conservation education initiatives, is vital for maintaining population stability and genetic diversity. Strategic habitat monitoring, coupled with public awareness efforts, will support sustainable management and help protect this endemic species from ongoing environmental and human-induced threats. These findings emphasise the importance of conservation strategies that account for restricted gene flow and unique habitat requirements of this endangered species.

## Figures and Tables

**Table 1:** Absolute (2C) and monoploid (1C*x*) genome size of *Alyssum moellendorfianum*.

			2C (pg)			1C*x* (pg)			1C*x* (Mbp)
Locality, ID	*N*_FCM_	Ploidy	Mean ± SD	Min–Max	CV (%)	Mean ± SD	Min–Max	CV (%)	Mean ± SD
Džepi, DZE	19	2*x*	1.60 ± 0.07	1.50–1.76	4.44	0.80 ± 0.04	0.75–0.88	4.60	784.38 ± 34.84
Spiljani, SP	20	2*x*	1.64 ± 0.10	1.44–1.83	5.86	0.82 ± 0.05	0.72–0.91	5.67	803.79 ± 47.38
Zagorice, ZA	20	2*x*	1.61 ± 0.07	1.36–1.70	4.64	0.81 ± 0.04	0.68–0.85	4.64	789.96 ± 36.77
Pirići, PI	20	2*x*	1.51 ± 0.05	1.42–1.63	3.16	0.76 ± 0.02	0.71–0.82	3.28	740.11 ± 23.31
Repovice, RE	20	2*x*	1.47 ± 0.04	1.38–1.60	2.98	0.74 ± 0.02	0.69–0.80	3.00	719.90 ± 21.26
Čičevo, CI	20	4*x*	3.19 ± 0.17	2.70–3.44	5.38	0.80 ± 0.04	0.68–0.86	5.37	779.05 ± 41.79
Sadine, SA	20	4*x*	3.23 ± 0.15	3.04–3.67	4.54	0.81 ± 0.04	0.76–0.92	4.62	790.11 ± 35.83
Dudle, DU	18	4*x*	3.10 ± 0.20	2.80–3.51	6.52	0.77 ± 0.05	0.70–0.88	6.49	757.87 ± 49.33
Borci, BOR	20	4*x*	2.96 ± 0.06	2.86–3.11	2.05	0.74 ± 0.02	0.72–0.78	2.09	724.02 ± 14.63
Dužani, DUZ	18	4*x*	2.92 ± 0.10	2.79–3.14	3.52	0.73 ± 0.03	0.70–0.79	3.53	714.50 ± 25.27

2*x*—diploid; 4*x*—tetraploid; SD—standard deviation; CV—coefficient of variation; Min–Max—minimal and maximal values for 2C and 1C*x* genome size. Population codes are consistently used in all tables and figures.

**Table 2:** Average values of genetic diversity parameters for five analysed loci per population.

Ploidy	Locality ID	A_N_	A_E_	AR	R	*p*(R)	H_E_	H_O_	F
2*x*	DZE	3.0	1.71	3.00	0.586	0.506	0.369	0.260	0.301
	SP	2.8	1.77	2.77	0.667	0.631	0.389	0.330	0.157
	ZA	3.4	2.47	3.40	0.725	0.563	0.494	0.350	0.192
	PI	4.0	2.54	4.00	0.620	0.566	0.462	0.300	0.276
	RE	3.6	2.52	3.60	0.694	0.557	0.509	0.350	0.200
4*x*	CI	4.6	2.84	4.45	0.612	0.224	0.601	0.640	0.129
	SA	5.4	2.81	4.87	0.588	0.334	0.554	0.510	0.400
	DU	5.4	3.38	5.06	0.628	0.287	0.645	0.610	0.278
	BOR	4.4	2.75	4.18	0.649	0.299	0.564	0.500	0.314
	DUZ	6.0	3.51	5.48	0.578	0.120	0.665	0.550	0.343

A_N_—number of detected alleles; A_E_—number of effective alleles; AR—allelic richness; R—the effective and detected number of alleles ratio; *p*(R)—statistical significance at *p* < 0.05; H_E_—expected heterozygosity; H_O_—observed heterozygosity; F—inbreeding coefficient. Population codes correspond to those given in Table 1.

**Table 3:** Genetic diversity parameters for total sample per loci.

Loci	A_N_	A_E_	AR	R	*p*(R)	H_E_	H_O_	F	iMAF	*p*(iMAF)	F_IT_	F_IS_	F_ST_
AP34461	5	1.59	4.04	0.318	0.045 *	0.369	0.338	0.342	0.255	0.007 *	0.3467	0.2943	0.0744
AP31679	7	3.49	5.46	0.499	0.017 *	0.713	0.751	−0.047	0.397	0.041	−0.0397	−0.1149	0.0675
AP31733	6	2.26	3.91	0.377	0.044 *	0.558	0.353	0.572	0.322	0.020	0.5777	0.5102	0.1377
AP31640	10	1.68	6.45	0.168	0.003 *	0.406	0.388	0.091	0.131	0.001 *	0.1041	−0.0450	0.1427
AP10368	13	5.34	8.60	0.411	0.005 *	0.813	0.318	0.718	0.265	0.009 *	0.7211	0.6870	0.1089
Average	8.2	2.87	5.69	0.354	0.022	0.572	0.430	0.360	0.274	0.015	0.3668	0.2922	0.1055

A_N_—number of detected alleles; A_E_—number of effective alleles; AR—allelic richness; R—the effective and detected number of alleles ratio; *p*(R)—statistical significance at *p* < 0.05; H_E_—expected heterozygosity; H_O_—observed heterozygosity; F—inbreeding coefficient; iMAF—major allele frequency index; *p*(iMAF)—statistical significance at *p* < 0.01; * statistically significant values of *p*(R) and *p*(iMAF) at particular loci; F_IT_—the inbreeding coefficient of an individual relative to the total population. F_IS_—the inbreeding coefficient of an individual relative to its subpopulation. F_ST_ —the level of genetic differentiation among subpopulations.

**Table 4:** Principal components (PC) revealed by the PCA and correlation factors with the first discriminant function (DF) for the *Alyssum moellendorfianum* populations.

No.	Trait	PC1	PC2	DF1
1	Stem length	0.295	−0.137	0.064
2	8th cauline leaf length	0.345	−0.204	0.006
3	8th cauline leaf width	0.308	−0.283	-
4	15th cauline leaf length	0.332	−0.306	-
5	15th cauline leaf width	0.280	−0.300	-
6	8th and 15th cauline leaf distance	0.211	0.088	-
7	Petal length	0.285	0.346	−0.210
8	Petal width	0.210	0.259	-
9	Petal sinus deepness	0.127	0.161	−0.491
10	Sepal length	0.286	0.313	−0.580
11	Filament length	0.318	0.272	−0.428
12	Style length	0.252	0.311	−0.443
13	Trichome ray length on lower surface of middle cauline leaf	0.177	0.060	−0.038
14	Trichome density on lower surface	−0.080	0.281	−0.168
15	Trichome coverage on lower surface	−0.049	0.006	-
16	Trichome density on upper surface	−0.138	0.313	0.100
17	Trichome coverage on upper surface	−0.129	0.075	0.239

**Table 5:** Morphometric parameters for the polar axis (P) and equatorial diameter (E).

	Diploids						Tetraploids					
	PI		ZA		SP		BOR		SA		DUŽ	
	P	E	P	E	P	E	P	E	P	E	P	E
Min–Max	27.5− 32.5	25.0− 27.5	27.5− 32.5	22.5− 30.0	25.0− 32.5	25.0− 27.5	32.5− 36.25	27.5− 32.5	35.0− 38.7	27.5− 31.5	35.0− 40.0	27.5− 32.5
Mean ± SD	30.9 ± 1.7	25.6 ± 1.1	29.9 ± 1.7	25.8 ± 1.9	29.6 ± 2.3	25.1 ± 1.3	34.7 ± 1.1	29.8 ± 1.0	36.7 ± 1.2	30.0 ± 0.7	37.5 ± 1.4	31.2 ± 1.5
CV (%)	5.6	4.1	5.7	7.2	7.7	5.1	3.1	3.5	3.4	2.3	3.7	4.8

Min–max—minimal and maximal values; Mean ± SD—mean values with standard deviation; CV—coefficient of variation.

**Table 6:** List of *Alyssum moellendorfianum* sampled populations and number of analysed individuals.

No.	Locality, ID	Latitude/Longitude	Altitude	N_FCM_	N_STR_	N_AFLP_	N_cp_	N_m_	Voucher
1.	Čičevo, CI	43.513780/18.080581	454 m	20	20	5	4	20	53495
2.	Sadine, SA	43.490831/18.148128	668 m	20	20	5	5	20	53496
3.	Dudle, DU	43.540567/18.121261	1034 m	18	20	5	4	20	53497
4.	Borci, BOR	43.573517/18.022017	711 m	20	20	5	5	20	53503
5.	Dužani, DUZ	43.509894/18.152114	830 m	18	20	5	3	20	53504
6.	Džepi, DZE	43.675506/18.011992	757 m	19	20	5	4	19	53498
7.	Spiljani, SP	43.625247/18.021678	509 m	20	21	5	4	20	53499
8.	Zagorice, ZA	43.647475/17.987317	668 m	20	20	5	4	17	53500
9.	Pirići, PI	43.708400/17.981864	623 m	20	20	5	5	19	53501
10.	Repovice, RE	43.659747/17.964069	338 m	20	20	5	4	20	53502

ID—population identification code; sample size for FCM measurements (N_FCM_), microsatellites (N_STR_), AFLPs (N_AFLP_), chloroplast region sequences (N_cp_), and morphometry (N_m_).

## Data Availability

The data presented in this study are available within the article and Supplementary Materials.

## Supplementary Materials

The following supporting information can be downloaded at: https://www.mdpi.com/article/10.3390/plants14020146/s1, Table S1: Comparative analysis of the genetic diversity parameters of the studied populations for individual loci; Table S2: The pF_ST_ genetic differentiation matrix between 10 populations of *A. moellendorfianum*; Table S3: Primer sequences used in this study according to Sobczyk et al., 2017; Table S4: Differences observed in concatenated sequences of *rpl32-trnL* and *rpoB-trnC* on the studied samples of *Alyssum moellendorfianum*. Numbers 1–13 correspond to the detected haplotypes; Table S5: Average values of measured characters for 10 analysed populations; Table S6; Descriptive parameters for the pollen grain viability; Figure S1: Neighbor-joining analysis of AFLP data for 10 populations of *Alyssum moellendorfianum*, differentiated by ploidy level; Figure S2: Grouping of analysed sequences based on pairwise distances across 10 populations, without differentiation by ploidy level.
