# Supplementary material for: Spatial Distribution of Genetic, Ploidy, and Morphological Variation of the Edaphic Steno-Endemic Alyssum moellendorfianum (Brassicaceae) from the Western Balkans
Source: Plants (Basel). 2025 Jan 7;14(2):146. doi: 10.3390/plants14020146 (PMC11769202; doi:10.3390/plants14020146)

Supplementary Table S1. Comparative analysis of the genetic diversity parameters of the five studied populations for individual loci.

	Loci	A_N	A_E	AR	R	*p*(R)	[illegible]$_E$	H_O	F
CI,	AP34461	3	1.65	2.95	0.550	0.328	394	0.450	0.175
4*x*	AP31679	5	3.84	4.99	0.768	0.327	740	1.000	-0.339
	AP31733	5	2.86	4.98	0.572	0.093	651	0.800	0.020
	AP31640	5	1.89	4.53	0.379	0.035*	472	0.550	0.138
	AP10368	5	3.96	4.83	0.792	0.341	748	0.400	0.653
	**Average**	**4.6**	**2.84**	**4.45**	**0.612**	**0.224**	**601**	**0.640**	**0.129**
SA,	AP34461	3	1.23	2.51	0.410	0.360	186	0.100	0.768
4*x*	AP31679	6	2.85	5.79	0.475	0.045*	650	0.800	-0.079
	AP31733	2	2.00	2.00	1.000	0.900	506	0.350	0.708
	AP31640	9 [P]	2.59	7.59	0.288	0.014*	613	0.650	0.094
	AP10368	7 [P]	5.37	6.45	0.767	0.351	814	0.650	0.512
	**Average**	**5.4**	**2.81**	**4.87**	**0.588**	**0.334**	**554**	**0.510**	**0.400**
DU,	AP34461	3	1.85	2.61	0.617	0.316	461	0.350	0.651
4*x*	AP31679	6	4.53	5.66	0.755	0.650	780	1.000	-0.290
	AP31733	3	1.81	2.95	0.603	0.328	447	0.450	0.355
	AP31640	8	4.13	7.53	0.516	0.045*	758	0.900	-0.107
	AP10368	7	4.56	6.56	0.651	0.100	781	0.350	0.785
	**Average**	**5.4**	**3.38**	**5.06**	**0.628**	**0.287**	**645**	**0.610**	**0.278**
BOR,	AP34461	1	1.00	1.00	1.000	0.900		-	-
4*x*	AP31679	5	2.56	4.67	0.512	0.076	610	0.850	-0.323
	AP31733	3	1.72	2.61	0.573	0.348	418	0.350	0.591
	AP31640	4	1.60	3.78	0.400	0.089	374	0.400	0.218
	AP10368	9 [P]	6.85	8.84	0.761	0.086	854	0.400	0.770
	**Average**	**4.4**	**2.75**	**4.18**	**0.649**	**0.299**	**564**	**0.500**	**0.314**
DUZ,	AP34461	5	1.80	4.47	0.360	0.041*	445	0.450	0.259
4*x*	AP31679	5	2.67	4.90	0.534	0.078	626	0.800	-0.116
	AP31733	4	2.91	3.57	0.728	0.334	657	0.250	0.852
	AP31640	7	4.41	6.61	0.630	0.101	773	0.850	0.000
	AP10368	9	5.75	7.85	0.639	0.047*	826	0.400	0.722
	**Average**	**6.0**	**3.51**	**5.48**	**0.578**	**0.120**	**665**	**0.550**	**0.343**
DZE,	AP34461	3	1.52	3.00	0.507	0.320	344	0.200	0.424
2*x*	AP31679	5	2.63	5.00	0.526	0.077	620	0.500	0.198
	AP31733	2	1.42	2.00	0.710	0.900	296	0.050	0.835
	AP31640	2	1.17	2.00	0.585	0.900	142	0.150	-0.056
	AP10368	3	1.80	3.00	0.600	0.337	445	0.400	0.103
	**Average**	**3.0**	**1.71**	**3.00**	**0.586**	**0.506**	**369**	**0.260**	**0.301**
SP,	AP34461	3	1.82	2.95	0.607	0.359	452	0.620	-0.383
2*x*	AP31679	3	2.48	3.00	0.827	0.900	598	0.570	0.046

	**Loci**	**A_N**	**A_E**	**AR**	**R**	***p*(R)**	**[illegible]E**	**H_O**	**F**
	AP31733	2	2.00	2.00	1.000	0.900	511	0.380	0.259
	AP31640	2	1.05	1.95	0.525	0.900	048	0.050	0.000
	AP10368	4	1.51	3.95	0.378	0.100	338	0.050	0.862
	**Average**	**2.8**	**1.77**	**2.77**	**0.667**	**0.631**	**389**	**0.330**	**0.157**
ZA,	AP34461	3	1.45	3.00	0.483	0.352	309	0.350	-0.137
2*x*	AP31679	5	3.75	5.00	0.750	0.341	734	0.700	0.048
	AP31733	2	1.97	2.00	0.985	0.900	492	0.300	0.397
	AP31640	2	1.23	2.00	0.615	0.900	184	0.200	-0.086
	AP10368	5	3.96	5.00	0.792	0.326	749	0.200	0.738
	**Average**	**3.4**	**2.47**	**3.40**	**0.725**	**0.563**	**494**	**0.350**	**0.192**
PI,	AP34461	5	1.97	5.00	0.394	0.040*	494	0.400	0.194
2*x*	AP31679	5	2.87	5.00	0.574	0.091	653	0.700	-0.075
	AP31733	2	1.42	2.00	0.710	0.900	296	0.150	0.500
	AP31640	2	1.05	2.00	0.525	0.900	050	0.050	0.000
	AP10368	6	5.39	6.00	0.898	0.900	815	0.200	0.759
	**Average**	**4.0**	**2.54**	**4.00**	**0.620**	**0.566**	**462**	**0.300**	**0.276**
RE,	AP34461	2	1.56	2.00	0.780	0.900	358	0.450	-0.267
2*x*	AP31679	5	3.75	5.00	0.750	0.302	735	0.600	0.187
	AP31733	4	2.89	4.00	0.722	0.340	655	0.450	0.319
	AP31640	2	1.11	2.00	0.555	0.900	097	0.100	-0.027
	AP10368	5	3.31	5.00	0.662	0.345	699	0.150	0.790
	**Average**	**3.6**	**2.52**	**3.60**	**0.694**	**0.557**	**509**	**0.350**	**0.200**

A_N – number of alleles; A_E – number of effective alleles; AR – allelic richness; R – the ratio of effective and detected number of alleles; *p*(R) – statistical significance at $p<0.05$; H_E – expected heterozygosity; H_O – observed heterozygosity; F – inbreeding coefficient; ^{P} – private allele detected; * – statistical significance

Supplementary Table S2. The pF_{ST} genetic differentiation matrix between 10 populations of *A. moellendorfianum.*

	CI	SA	DU	BOR	DUZ	DZE	SP	ZA	PI
SA	0.0204								
DU	0.0946	0.0777							
BOR	0.1150	0.0796	0.2158						
DUZ	0.0537	0.0264	0.0716	0.1314					
DZE	0.1995	0.2086	0.2535	0.0908	0.2225				
SP	0.1134	0.1049	0.2790	0.2489	0.1533	**0.4289**			
ZA	0.0395	0.0398	0.1520	0.0858	0.0848	0.1397	0.1247		
PI	0.1120	0.1220	0.2415	0.0451	0.1436	0.0732	0.1556	0.0657	
RE	0.0281	0.0509	0.1627	0.1498	0.0950	0.2344	0.0325	0.0252	0.0877

Supplementary Table S3. Primer sequences from Sobczyk et al., 2017 used in this study.

STR loci	Primer sequence 5' → 3'	Colour	Range (bp)	N_A	Range (bp)	N_A*
AP31733*	F: CCGATTCCCAAAGATCCCGTG	6-FAM	187–202	5	169–199	6
	R: GGTATTGCCGCAAAGTTTTTCA					
AP32282	F: TCCTCCTCACTTTCGCTGAA	6-FAM	294–315	5	-	-
	R: GACGGTTGATGGCGGTTTTG					
AP31679*	F: TCCTCACCAAAGCTCAGCG	HEX	363–399	12	291–327	7
	R: CTTAGCCTCCTCCTCCCTCT					
AP801	F: TGGAGGTGGGATATGAGCAAA	HEX	208–229	5	-	-
	R: CGAGCAGAGGAGACCAAAGA					
AP31640*	F: CGAAAACTCTGCGATGTGGC	HEX	253–292	10	225–255	10
	R: CGTCTCTTGGGTTTTGCTGC					
AP34461*	F: AAGGGAAAGTCAGAAGCAGAGC	6-FAM	147–168	7	112–130	5
	R: AGTTCTTCAAAGTTTCATAGACAACA					
AP10368*	F: TCTAACTGACGGAGGGGTTG	6-FAM	343–355	4	343–385	13
	R: TGCCATTCTTGAAGCACTGC					
AP5386	F: GGGATTCTTGGTCGGCTCAA	HEX	373–385	5	-	-
	R: CTGATGGAGATGCTTGTGGGT					

*Loci amplified in *Alyssum moellendorfianum*; N_A- number of alleles amplified in *Alyssum serpyllifolium*; N_A* - number of alleles amplified in *Alyssum moellendorfianum*

Supplementary Table S4. Differences observed in concatenated sequences of *rpl32-trnL* and *rpoB-trnC* on the studied samples of *Alyssum moellendorfianum*. Numbers 1-13 correspond to the detected haplotypes.

1	G	T	G	T	T	G	T	T	T	G	G	T	A	T	A	T	T	C	A	A	A	T	C	T	C	T	A	A
2	.	G	A	C	.	.	.	.	.	T	.	.	.	A	T	.	.	.	.	.	.	.	.	.	A	.	C	G
3	.	.	.	.	A	.	.	.	A	T	.	.	.	.	.	.	.	A	.	.	.	.	.	.	A	.	C	.
4	.	.	.	.	A	.	.	.	A	.	.	.	.	.	.	.	.	A	.	.	.	.	.	.	A	.	C	.
5	.	.	.	.	.	.	.	.	A	T	.	.	.	.	.	.	.	A	.	.	.	.	.	.	A	.	C	.
6	.	G	A	C	.	.	.	.	.	T	.	.	.	.	T	C	G	.	.	.	.	.	.	.	A	.	C	G
7	A	.	.	C	.	T	.	.	.	T	.	.	.	.	.	.	.	.	C	.	.	G	.	.	A	.	C	G
8	.	G	A	C	.	.	.	.	.	T	.	.	.	.	T	.	.	.	.	.	.	.	.	.	A	.	C	G
9	.	.	.	.	.	.	.	.	.	.	.	.	.	.	.	.	.	A	.	.	.	.	.	.	.	.	.	.
10	.	.	.	C	.	.	.	G	.	T	.	.	.	.	.	.	.	.	.	.	T	.	T	.	A	A	C	.
11	.	.	.	C	.	.	.	.	.	T	.	.	.	.	.	.	.	.	.	C	.	.	.	A	A	.	C	.
12	.	.	.	.	.	.	.	.	.	.	.	C	C	.	.	.	.	A	.	.	.	.	.	.	.	.	.	.
13	.	.	.	C	.	.	G	.	.	T	A	.	.	.	.	.	.	.	.	.	.	.	.	.	A	.	C	.

Supplementary Table S5. Average values of measured characters for 10 analysed populations.

	DU	DUZ	SA	BOR	CI	DZE	SP	ZA	PI	RE
1	69.30	87.40	74.50	79.70	65.85	61.94	71.15	63.88	95.21	96.60
2	6.33	6.07	5.97	6.81	6.18	5.82	5.85	5.58	7.22	6.90
3	1.56	1.38	1.55	1.78	1.29	1.55	1.38	1.38	1.70	1.95
4	5.84	5.35	5.22	6.36	5.33	4.93	5.53	4.58	6.17	6.91
5	1.89	1.76	2.26	2.50	1.84	1.93	1.76	1.62	1.95	2.92
6	17.44	19.00	14.49	12.89	12.28	13.15	9.83	14.81	19.92	15.22
7	5.08	5.41	5.07	4.85	4.22	4.58	4.66	4.9	4.79	4.64
8	2.59	2.96	2.17	2.14	1.74	2.24	2.12	1.98	1.95	2.37
9	0.23	0.34	0.33	0.27	0.31	0.19	0.23	0.24	0.25	0.23
10	3.15	3.51	3.42	3.27	3.17	3.01	3.09	3.09	3.03	3.08
11	3.73	3.94	3.94	3.75	3.37	3.32	3.23	3.59	3.67	3.35
12	2.37	2.67	2.84	2.25	2.16	2.11	2.05	2.06	2.20	2.05
13	0.18	0.21	0.21	0.19	0.18	0.19	0.19	0.21	0.20	0.19
14	21.85	19.65	17.85	16.20	19.6	18.31	17.7	17.82	19.84	17.25
15	2.85	2.85	2.85	2.65	2.70	3.00	3.00	2.88	2.79	2.95
16	16.40	16.10	15.50	13.10	14.60	14.78	16.2	15.88	16.26	14.7
17	2.35	2.45	2.15	2.15	2.10	2.47	2.45	2.18	2.26	2.35

1-stem length; 2- 8th cauline leaf length; 3- 8th cauline leaf width; 4- 15th cauline leaf length; 5- 15th cauline leaf width; 6- 8th and 15th cauline leaf distance; 7- petal length; 8- petal width; 9- petal sinus deepness; 10- sepal length; 11- filament length; 12- style length; 13- trichome ray length on lower surface of middle cauline leaf; 14- trichome density on lower surface; 15- trichome coverage on lower surface; 16- trichome density on upper surface; 17- trichome coverage on upper surface. Characters 1-13 are in mm. Characters 14 and 16 represent a number of trichomes on the area of 0.5 mm^2 with homogenous indumentum not including median vein of middle cauline leaf. Characters 15 and 17 represent coverage of trichomes of middle cauline leaf with following measures: 0: 0-33%; 1: 33-66%; 2: 66-95% and 3: 95-100% coverage.

Supplementary Table S6. Descriptive parameters for the pollen grain viability.

	Diploids					Tetraploids				
	DZE	SP	ZA	PI	RE	CI	SA	DUZ	DU	BO
Min-max	97-100	93-99	86-100	60-95	70-97	90-96	85-96	98-100	95-100	98-100
Mean± SD	99.00± 1.22	96.60± 2.50	94.80± 5.74	86.80± 15.10	89.60± 11.23	92.60± 2.30	91.20± 3.90	99.20± 0.83	98.00± 1.87	98.80± 0.83
CV (%)	1.23	2.59	6.08	17.40	12.60	2.46	4.34	0.84	1.90	0.84

Min-max – minimal and maximal values (%); Mean± SD – mean values (%) with standard deviation; CV – coefficient of variation

Supplementary Figure S1. Neighbor-Joining analysis of AFLP data for 10 populations of *Alyssum moellendorfianum*, differentiated by ploidy level.

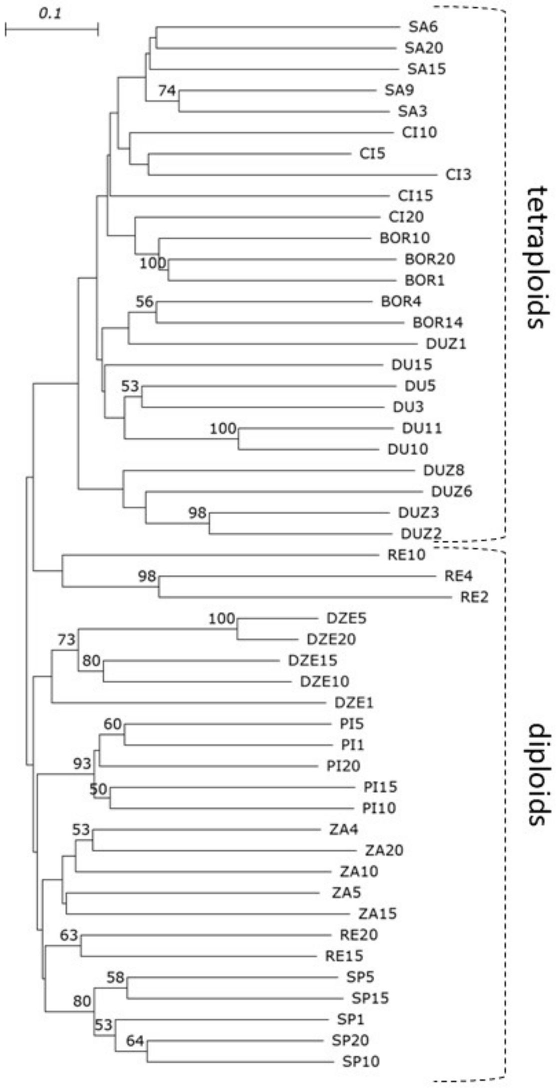

Supplementary Figure S2. Neighbor-Joining tree based on pairwise distances of analyzed sequences across 10 populations.

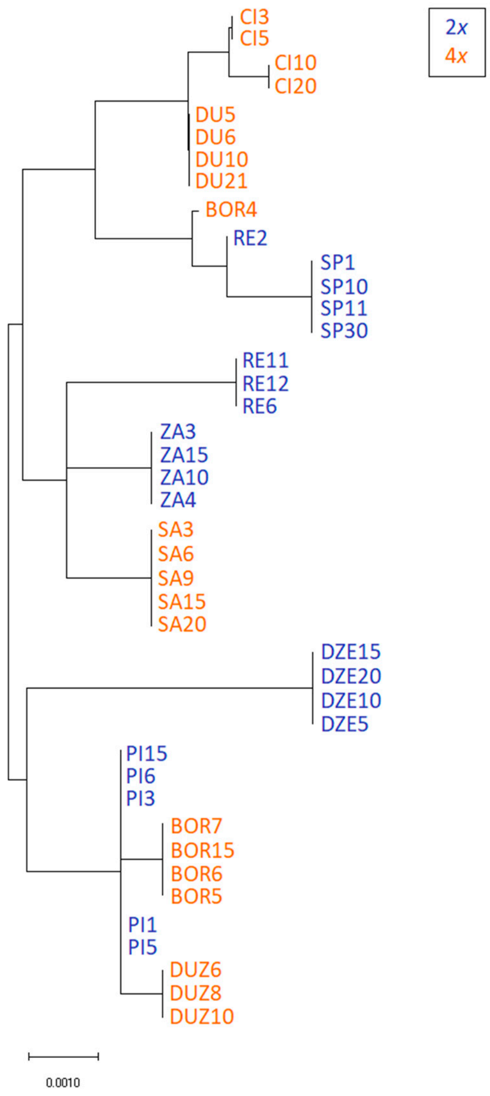

Supplement: Supplementary file 1 [file plants-14-00146-s001.zip › plants-3381001-supplementary.pdf]
